# Supplementary material for: The impact of Traditional Chinese Medicine utilization on life expectancy and mortality
Source: PLoS One. 2025 Dec 4;20(12):e0337593. doi: 10.1371/journal.pone.0337593 (PMC12677513; doi:10.1371/journal.pone.0337593)
Supplement: S1 File — (PDF) [file pone.0337593.s001.pdf]

S1 File. Supplementary methods

We described the details of the rolling extrapolation algorithm in the following four major steps. First, each individual of a study cohort was given a weight equal to inverse probability of the individual being in the cohort which is estimated by a multinomial logistic regression model with measured confounding covariates. That is, each study cohort is inflated with the weights of the participants to form a pseudo-cohort in which confounders are equally distributed across the pseudo-cohorts. The adjusted Kaplan-Meier estimator (AKME) was applied to the weighted survival data of individuals in the study cohort to obtain the survival function, denoted by $S(t)$, for the cohort. We may call $S(t)$ the confounder-adjusted survival function of the study cohort. Second, Monte Carlo methods were used to generate survival times of referents whose age and sex matched with participants in the study cohort using life tables of general population. The same weights were assigned for the referents matched with the participants in the study cohort. The confounder-adjusted lifetime survival function of the reference population was obtained by applying AKME on the weighted survival times of the generated referents and denoted by $S_r(t)$. We renamed $S_r(t)$ as $S_p(t)$ for use as a relevant predictor in extrapolating the survival function of the study cohort when $S_r(t) > S(t)$ during the follow-up period. If $S_r(t) \leq S(t)$, a proper hazard value δ was subtracted from the reference population to ensure the

predictor $S_p(t) = S_r(t) \times e^{\delta t} > S(t)$. The relative survival function, $W(t) = \frac{S(t)}{S_p(t)}$, is then within 0 and 1. Third, the logit transformation of the relative survival would straighten the curve $\text{logit}[W(t)]$ which is relatively easy to be extrapolated. Fourth, we used restricted cubic spline models to fit $\text{logit}[W(t)]$ for the observed time period $t = 1,2, \ldots, F$ months. The fitted curve was used to predict the $\text{logit}[W(t)]$ one month ahead. The predicted $\text{logit}[W(F+1)]$ was usually quite accurate due to the approximate linearity property of $\text{logit}[W(t)]$ and treated as an "observation" at month $F+1$. We then repeated the extrapolation procedures by rolling the same-length observation periods one month ahead, $t = 2, 3, \ldots, F+1$, and refit the restricted cubic spline models for the updated observation periods to predict the value of $\text{logit}[W(F+2)]$. By repeatedly performing the above procedures of extrapolating $\text{logit}[W(t)]$ month-by-month to a time L beyond which all subjects of the cohort died, we could then invert transformation of the extrapolated $\text{logit}[W(t)]$ to obtain estimate of relative survival function $\widehat{W}(t)$ and lifetime survival function $\hat{S}(t) = \widehat{W}(t) \times S_p(t)$ of the cohort. The confounder-adjusted LE of each study cohort was obtained by summing the extrapolated confounder-adjusted lifetime survival function, $LE = \sum_{t=0}^{L} S(t)$. The loss or gain of LE of frequent TCM user cohort is estimated by the difference of confounder-adjusted LE between the cohort of frequent and non-frequent TCM users.
