# Supplementary material for: The impact of Traditional Chinese Medicine utilization on life expectancy and mortality
Source: PLoS One. 2025 Dec 4;20(12):e0337593. doi: 10.1371/journal.pone.0337593 (PMC12677513; doi:10.1371/journal.pone.0337593)
Supplement: S1 Table — (PDF) [file pone.0337593.s002.pdf]

S1 Table: ICD codes for baseline comorbidities

**Comorbidities**	***ICD-9-CM***	***ICD-10-CM***
Cardiovascular disease	390－459	I00－I99
Diabetes mellitus	250	E10－E14
Chronic lung diseases (asthma or chronic obstructive pulmonary disease)	490－496	J40－J47
Chronic kidney diseases	585	N18
Chronic liver disease	571	K70－K77
Dementia	290－294	F00－F03
