# Supplementary material for: The impact of Traditional Chinese Medicine utilization on life expectancy and mortality
Source: PLoS One. 2025 Dec 4;20(12):e0337593. doi: 10.1371/journal.pone.0337593 (PMC12677513; doi:10.1371/journal.pone.0337593)
Supplement: S2 Table — (PDF) [file pone.0337593.s003.pdf]

S2 Table. Hazard Ratios (HRs) and 95% CIs for associations between TCM utilization (frequent user vs. non-frequent user) and risk of major chronic diseases development

Disease outcomes	TCM utilization	HR (95%CI) [a]	p-value
Cardiovascular disease	Non-frequent TCM user	1.00	
	Frequent TCM user	0.96 (0.86, 1.08)	0.5196
Diabetes mellitus	Non-frequent TCM user	1.00	
	Frequent TCM user	0.96 (0.84, 1.09)	0.5436
Chronic lung diseases	Non-frequent TCM user	1.00	
	Frequent TCM user	0.99 (0.87, 1.13)	0.8816
Chronic kidney diseases	Non-frequent TCM user	1.00	
	Frequent TCM user	1.03 (0.87, 1.22)	0.7336
Chronic liver disease	Non-frequent TCM user	1.00	
	Frequent TCM user	0.91 (0.74, 1.11)	0.3444
Dementia	Non-frequent TCM user	1.00	
	Frequent TCM user	0.86 (0.72, 1.02)	0.0778

[a] Cox model adjusted for age, sex, enrolment year, education level, marital status, monthly household income, employment status, lifestyle factors, and medical history of cardiovascular disease, diabetes mellitus, chronic lung diseases, chronic kidney diseases, chronic liver disease, and dementia.
