# Supplementary material for: The impact of Traditional Chinese Medicine utilization on life expectancy and mortality
Source: PLoS One. 2025 Dec 4;20(12):e0337593. doi: 10.1371/journal.pone.0337593 (PMC12677513; doi:10.1371/journal.pone.0337593)
Supplement: S3 Table — (PDF) [file pone.0337593.s004.pdf]

S3 Table. Hazard Ratios (HRs) and 95% CIs for associations between TCM utilization (per 10% of TCM utilization increase) and risk of major chronic diseases development

Disease outcomes	TCM utilization (time-varying)	HR (95%CI) [a]	p-value
Cardiovascular disease	per 10% of TCM utilization increase	0.98 (0.97, 0.99)	<0.0001
Diabetes mellitus	per 10% of TCM utilization increase	0.98 (0.97, 0.99)	<0.0001
Chronic lung diseases	per 10% of TCM utilization increase	1.01 (1.00, 1.01)	0.0658
Chronic kidney diseases	per 10% of TCM utilization increase	0.99 (0.98, 1.00)	0.0242
Chronic liver disease	per 10% of TCM utilization increase	0.98 (0.97, 0.99)	0.0003
Dementia	per 10% of TCM utilization increase	0.97 (0.96, 0.98)	<0.0001

[a] Cox model adjusted for age, sex, enrolment year, education level, marital status, monthly household income, employment status, lifestyle factors, and medical history of cardiovascular disease, diabetes mellitus, chronic lung diseases, chronic kidney diseases, chronic liver disease, and dementia.
